# The Local Atomic Structure of Colloidal Superparamagnetic Iron Oxide Nanoparticles for Theranostics in Oncology

**DOI:** 10.3390/biomedicines6030078

**Published:** 2018-07-18

**Authors:** Elena Kuchma, Stanislav Kubrin, Alexander Soldatov

**Affiliations:** 1 Smart Materials Research Center, Southern Federal University of Russia, 344006 Rostov-on-Don, Russia; stasskp@gmail.com; 2 Research Institute of Physics, Southern Federal University of Russia, 344006 Rostov-on-Don, Russia

**Keywords:** superparamagnetic iron oxide nanoparticles (SPION), theranostics in oncology, local atomic structure, XANES spectroscopy, Mössbauer spectroscopy

## Abstract

The paper contains an overview of modern spectroscopic methods for studying the local atomic structure of superparamagnetic nanoparticles based on iron oxide (SPIONs), which are an important class of materials promising for theranostics in oncology. Practically important properties of small and ultra small nanoparticles are determined primarily by their shape, size, and features of the local atomic, electronic, and magnetic structures, for the study of which the standard characterization methods developed for macroscopic materials are not optimal. The paper analyzes results of the studies of SPIONs local atomic structure carried out by X-ray absorption spectroscopy at synchrotron radiation sources and Mössbauer spectroscopy during the last decade.

## 1. Introduction

One of the modern trends in cancer treatment is the use of simultaneous diagnostics and therapy-theranostics [1]. Development of novel diagnostic tools combined with image control during the therapy opens a road for future precision medicine in cancer treatment [2]. Nanoparticles for theranostic application in oncology could be developed to support several types of diagnostic and therapy methods [3,4]. Moreover, advances in nanoscience make it possible to combine even three functions in one: namely targeting, diagnostics, and therapeutics to combat the cancer successfully. The major challenges in successful clinical translation of tumor specific nanoparticle delivery include overcoming various biological barriers and demonstrating enhanced therapeutic efficacy over the current standard of care in the clinic [5]. Important successful steps have been made in the last few years towards the development of new classes of nanoparticles that can respond to tumor microenvironmental conditions and successfully deliver therapeutic agents to cancer cells [6]. It is expected that theranostic agents can be designed to personalize treatment for precision medicine of cancer and minimize damage to normal tissue [7]. 

One of the most developed families of theranostic nanoparticles is superparamagnetic iron oxide nanoparticles (SPIONs) [8]. These magnetic nanoparticles based on iron oxides have recently attracted special attention of specialists in various fields: from chemistry [9] to nanomedicine [10] and ecology [11]. As in the case of other types of nanoparticles, the characteristics of magnetic nanoparticles important for theranostics applications depend first of all on their size (shape) and fine details of their local atomic structure [12]. The biocompatibility of nanoparticles significantly depends also on surface characteristics. For example, small (9.7 nm) crystalline iron oxide nanocubes having magnetite structure coated by silica are found to be not cytotoxic up to rather high concentrations of 100 µg Fe/mL [13]. One of the most promising classes of magnetic nanoparticles are small nanoparticles, the size of which is so small that the domain structure can no longer be realized and, accordingly, nanoparticles acquire a superparamagnetic character. Such superparamagnetic nanoparticles find their important application in the theranostics of cancer diseases [14]. 

Physicochemical properties of SPIONs determine their distinctive pharmacokinetics when encountering in vivo biological barriers [15]. Current clinical trials of SPION-based MRI (Magnetic Resonance Imagine) contrast agents shows that although gadolinium-based agents are still in dominant position of clinical MRI enhancement, one could expect that SPIONs will add more opportunities for both diagnosis and imaging guided therapy in the near future. Polypeptide labeled SPIONs are found to be very effective for targeting prostate cancer specific membrane antigen [16]. Several studies have shown that not only pure iron oxide magnetic nanoparticles are useful for theranostics applications. Comparative analysis is in progress to study the features of SPIONs with gadolinium loaded nanoparticles [17] and europium-doped gadolinium sulfide nanoparticles of about 8 nm diameter and spherical shape, which have NaCl crystal structure and high crystallinity [18]. It was found that holmium doping of magnetite SPIONs (with sizes between 8 and 15 nm) results in significant distortions of the nanocrystals originating from the comparative atomic radii of Ho and Fe atoms [19]. Europium engineered iron oxide nanocubes are also found to be effective for both T1 and T2 contrast MRI in living subjects [20] and it was found that their cytotoxicity characteristics are rather good. 

Ultra small and small SPIONs having size distribution between 2 and 12 nm can be obtained by using water-in-oil or oil-in-water micro-emulsion technique [21]. Biosafety of magnetite SPIONs have been established up to 200 µg/mL [22]. But, when the cells are exposed to very high doses of SPIONs, formation of excess Reactive Oxygen Species (ROS) takes place, affecting normal functioning of the cell and leading to apoptosis or cell death [23]. It was shown that integration of 100 nm × 10 nm hexagonal MoS_2_ nanoflakes with 5 nm cubic SPIONs opens a way for magnetically targeted (by MRI) photothermal cancer therapy [24]. 

Complementary imaging modalities provide much more information that either method alone can yield. Dual modality contrast agents for MRI and positron emission tomography (PET) could be prepared by ^64^Cu doped SPIONs [25]. The SPION core is found to be about 8 nm in size, having mostly magnetite structure with some admixture of maghemite phase iron oxides. ^64^Cu ions added during co-precipitation is in 2^+^ oxidation state, working in direct competition with Fe^2+^ ions to occupy the octahedral site in the Fe_3_O_4_ structure [26]. The shape of SPIONs is also an important characteristic that affects the biocompatibility of the nanoparticles. It was found that rod-shaped iron oxide nanoparticles are more toxic than sphere-shaped nanoparticles to murine macrophage cells [27].

**Figure 1:**
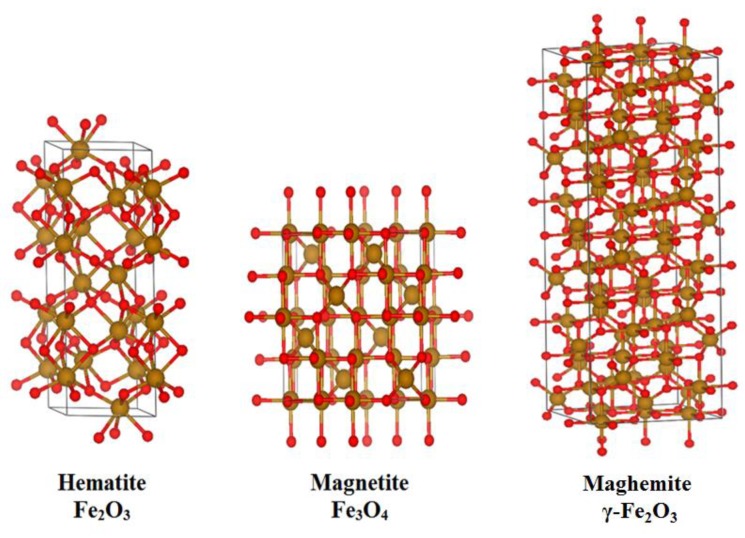
Possible crystal structures of the Superparamagnetic Iron Oxides Nanoparticles (SPIONs).

Earlier, reviews of characterization of magnetic nanoparticles by standard methods were conducted [28,29,30]. However, since the sizes of the SPIONs sometimes reach one or two nanometers, the use of standard (for example, x-ray or electron diffraction) methods becomes difficult, because the small size of the nanoparticles results in broad diffraction reflexes and, accordingly, diminishes the accuracy of determination of the atomic structure parameters. Crystal structures of iron oxide nanoparticles are presented in Figure 1. Because magnetite and maghemite structures are rather similar, their diffraction patterns are also very similar, especially in the case of rather small nanoparticles where the diffraction reflexes are not as narrow as in the macroscopic case.

Therefore, at the present time, spectroscopic methods start to be actively involved in the studies of SPIONs’ local atomic structure. It was found that spectroscopic methods could provide an extremely high sensitivity to the parameters of SPION local atomic structure. Among this group of nanocharacterization methods, an important place is occupied by X-ray absorption spectroscopy (mostly using synchrotron radiation sources) [31] and Mössbauer spectroscopy [32] as these methods could be applied to study materials even without long-range order in the atomic positions. Serious increasing of the X-ray radiation source’s intensity (synchrotron radiation facilities and x-ray free electron lasers) significantly improves the possibility to study extremely small amounts of nanoparticles (for example, low concentrated SPIONs in biological liquids and tissues). The possibility of high time resolution at the abovementioned sources of X-ray radiation enables one to study not only the nanomaterials themselves at static conditions, but also their transformations during the processes in which they are involved. 

## 2. Spectroscopic Methods for SPION Local Atomic Structure Studies

### 2.1. X-ray Absorption Spectroscopy

**Figure 2:**
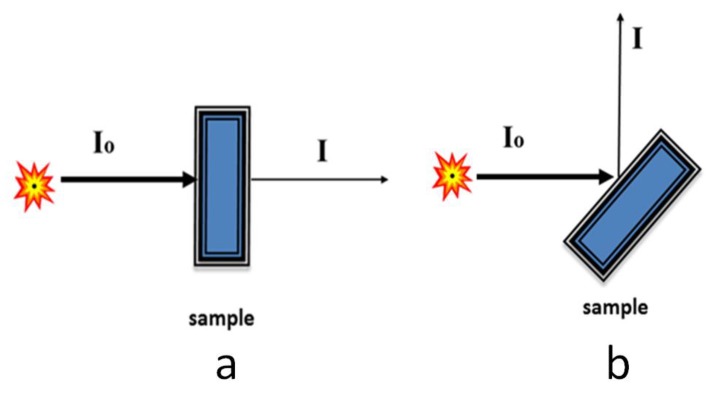
The scheme of the experiment for detection by two methods: true absorption (**a**) and fluorescence (**b**).

To study the local atomic and electronic structures of nanoparticles, a group of techniques that obtain structural information from the analysis of the shape of the X-ray absorption spectra has recently been very successful [33]. Such methods include studying the fine structure of X-ray absorption (the international term is X-ray Absorption Fine Structure—XAFS spectroscopy). The XAFS method is most often implemented in mega-class research facilities—synchrotron radiation sources of high intensity—so the experiment time is very short, which allows one to study a large number of samples and requires a small amount of a substance for analysis. Measurements of the X-ray absorption spectrum can be carried out in several ways. The “true” X-ray absorption is measured by determining the difference in the X-ray intensity before and after the sample (Figure 2a). However, such a direct method of measurement is not easy to realize, since it demands high homogeneity and low thickness of the samples under the study. More universal methods are based on the measurement of secondary processes: for example, X-ray fluorescence emitted after the absorption of the incoming X-ray radiation in the sample, or by measuring the total or partial yield of photoelectrons leaving the sample (Figure 2b).

**Figure 3:**
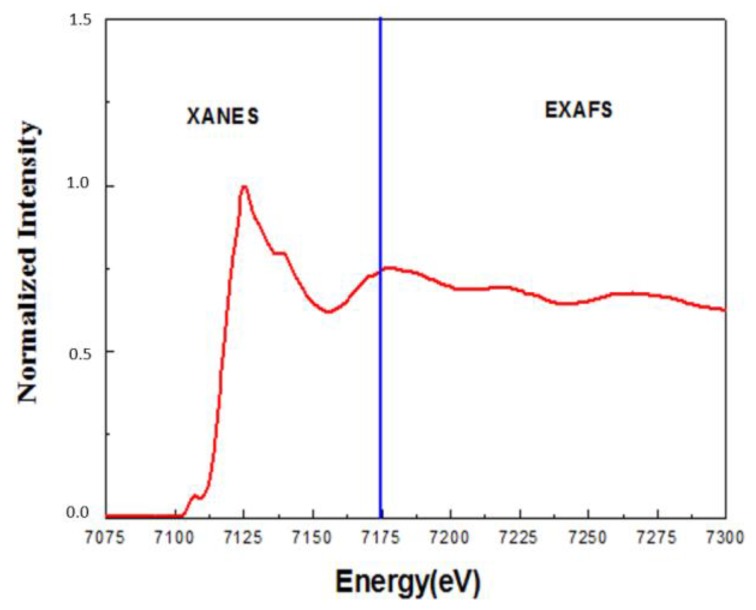
X-ray absorption spectrum of iron oxide nanoparticles (both XANES and EXAFS regions).

The choice of specific techniques is determined by the characteristics of the samples being studied and the specific tasks of the study. For XAFS measurements, the samples can be in any aggregate state [34], and can even directly analyze nanoparticle samples in biological tissues. This is extremely important for SPIONs, which are intended for the administration to biological tissues. Analysis of XAFS spectra allows obtaining information about the positions of atoms in the region of several coordination spheres around the X-ray excited atom, (i.e. it is possible to study bond lengths and bond angles) [35]. Typically, the XAFS spectrum is divided into two main areas: the region of the X-ray Absorption Near Edge Structure (XANES) and the Extended X-ray Absorption Fine Structure (EXAFS) (Figure 3). Although XANES and EXAFS are of close origin, they are normally treated separately, since the information that can be obtained from these two parts of the X-ray absorption spectrum is different [36] and the tools for the analysis of these two parts of X-ray absorption spectrum are different. For example, the change in the symmetry of the local environment of the atom (the angles of the chemical bond), even without changing the distance between the absorbing atom and its neighbors and the number of neighbors, leads to a change in the structure of the XANES spectrum, while the structure of the EXAFS spectrum remains unchanged. Thus, the EXAFS method in most cases cannot be used for the study of full 3D local atomic structure of materials without long-range order, while XANES might be useful for such a purpose.

EXAFS is successfully used to analyze the atomic structure (the radial distribution function of atoms around the X-ray absorbing atom) of nanoparticles up to small sizes of the order of 1 nm or less [37]. It is easier to get the available structural information on the basis of the EXAFS analysis (spectral region extending from about 100 eV behind the edge and above) than to analyze the XANES spectral region (extends from the absorption edge up to 100 eV above the edge) [38].

This is explained by the shorter mean free path of the photoelectron at energies corresponding to the region of EXAFS, which makes the signal from the single scattering of the photoelectron wave by the surrounding atoms predominant [39]. This is an advantage that makes the EXAFS spectra a more direct source of information on such important structural parameters as bond lengths and coordination numbers [40,41], however, the absence of multiple scattering of photoelectrons by neighboring atoms practically completely eliminates the possibility of determining such important parameters of the local atomic structure like the angles of chemical bonding. Thus, using the EXAFS method, it is almost impossible to determine the complete three-dimensional local structure of the material. On the other hand, the XANES region, which includes a near-edge region of about 100 eV behind the edge, is very sensitive to the local environment and the electronic structure of the X-ray absorbing atoms, in particular to bond angles, bond lengths, coordination numbers, and oxidation states of the atoms under study. However, the determination of a complete three-dimensional local atomic structure of a material without long-range order in the arrangement of atoms requires complex theoretical calculations using supercomputers [42]. Application of both XANES and EXAFS methods makes it possible to thoroughly study the local atomic structure and phase composition of the nanoparticles [43,44].

One of the tasks in the development of new techniques for the synthesis of small nanoparticles is the problem of determining the phase composition of the final product. When synthesizing iron oxide nanoparticles, a mixture of several phases (Fe_3_O_4_, γ-Fe_2_O_3_, α-Fe_2_O_3_) is often obtained. In the case of γ-Fe_2_O_3_, impurity defects formation is an especially important process [45]. Since magnetite (Fe_3_O_4_) and maghemite (γ-Fe_2_O_3_) crystallize in lattices of similar types (reverse spinel), it is very difficult to distinguish these two oxides by X-ray diffraction [45,46,47,48]. The diffraction pattern of Fe_3_O_4_ nanoparticles has almost the same set of reflexes as γ-Fe_2_O_3_ nanoparticles. X-ray diffraction methods do not allow unambiguous, accurate phase analysis of small nanoparticles, due to the size effect on the X-ray diffraction pattern [49,50]. The decrease in the size of the crystallites leads to a significant broadening of the diffraction reflexes [51,52,53,54]. Another cause of broadening is the presence of structural defects, which, as a rule, are quite numerous in nanoparticles (for example, lattice dislocations), leading to lattice deformations [55]. These factors lead to a smearing of the diffraction reflections, which complicates the interpretation of the diffractograms and lowers the accuracy of the analysis of the nanoparticles’ atomic structure. It should be noted that the presence of various kinds of structural defects in the sample leads to a change in the properties of the nanoparticles [55,56,57]. The effect of the ordering of vacancies in the B position of maghemite on the magnetic properties of nanoparticles was established [58]. In particular, an increase in the number of vacancies in the octahedral sublattice leads to a decrease in the magnetic moment of the particles [59,60]. Thus, to obtain nanoparticles of iron oxides with the required characteristics, it is necessary to study the defectiveness and fine details of the local atomic structure of nanoparticles. EXAFS and XANES methods, due to their high sensitivity to the coordination number and changes in the local atomic structure, are important tools for analyzing the phase composition of nanoparticles and the local atomic structure of iron oxide nanoparticles [61].

Since the XANES method makes it possible to also investigate the density of unoccupied electronic states above the Fermi level, it is sensitive to the oxidation state of the ion under investigation [62,63]. Thus, on the basis of a comparison of the energy position of the absorption edge in the material under study, with the spectra of reference samples with a known oxidation state of the ion, it is possible to determine the oxidation state of the ion in the material under study [64]. The position of the inflection point of the absorption edge is determined by the maximum of the first derivative at this point [65,66,67]. A similar analysis was carried out for two reference samples Fe_3_O_4_ and γ-Fe_2_O_3_ by the XANES method near the K-edge of Fe ions [68]. Magnetite (Fe_3_O_4_) contains Fe^2+^ ions (33%) in the B position and Fe^3+^ ions (67%) in positions A and B. Iron ions in maghemite (γ-Fe_2_O_3_) are only in the Fe^3+^ state. Thus, a different chemical shift of the main absorption edge is observed in the XANES spectra for Fe_3_O_4_ and γ-Fe_2_O_3_ compounds. Comparison of the energy positions of experimental XANES peaks (7185, 7226, and 7275 eV) and their intensities in the spectrum of the synthesized SPIONs with the reference ones made it possible to establish that the synthesized sample has a local atomic structure analogous to the γ-Fe_2_O_3_ phase of macroscopic iron oxide [64]. A similar study of the local atomic structure, carried out in Ref. [69], also made it possible to establish exactly the phase composition of the iron oxide nanoparticles. In this study, the XANES method was used to obtain information on the local atomic structure of synthesized nanoparticles, and it was established that the structure of nanoparticles is similar to the structure of macroscopic maghemite (γ-Fe_2_O_3_).

The use of both XANES and EXAFS methods is an important addition to the X-ray diffraction method for small nanoparticles’ structure characterization. Thus, although the X-ray diffraction of Fe_3_O_4_ nanoparticles synthesized by the plasma synthesis method showed that the impurity phase of γ-Fe_2_O_3_ is present in the sample, most spectral features of the nanoparticles are close to the spectrum of the crystalline Fe_3_O_4_ reference sample [70]. The near-peak singularities of the Fe K-edge are sensitive to the local geometry (in particular, to the coordination of iron atoms by oxygen atoms). XANES also provided information on the oxidation state of iron ions in nanoparticles. The use of EXAFS spectroscopy made it possible to determine the radial distribution function of atoms and to obtain information about local disorder (Debye-Waller factor). The obtained data made it possible to simulate the distribution of oxygen atoms around iron atoms and unambiguously establish that the synthesized nanoparticles possess a magnetite structure [70].

In Ref. [71], the brick-like nanoparticles (BLN) of Ag@Fe_3_O_4_ type formed by a silver core surrounded by a shell of Fe_3_O_4_ (magnetite) were investigated. It was assumed that the envelope of magnetite prevents the diffusion of silver ions into the environment, increasing the biocompatibility of these nanoparticles. The TEM image analysis showed that the structure of nanoparticles differs from the traditionally created core-shell structures, since Ag@Fe_3_O_4_ nanoparticles have a quasi-cubic shape with a size of 13.3 nm. The oxidation state of iron ions was studied using the XANES method. The XANES shape of the Fe K-edge was close to those observed in macroscopic magnetite. The energy position of the spectrum peaks corresponds to the octahedral ions (Fe^3+^ and Fe^2+^). In the BLN sample, the peaks are shifted to higher energies. This corresponds to nanometer samples containing some fraction of maghemite. From the XANES spectra analysis, it was found that the average oxidation state of iron for the BLN sample indicates that most of the iron oxide phase is indeed a magnetite one. A small amount of maghemite phase was also observed. The position of the main XANES peak (which is sensitive to the oxidation state) is slightly shifted towards higher energies for these nanoparticles, as expected for more oxidized types of iron oxides.

In some cases, for the practical application of iron oxide-type nanoparticles of the “core-shell” type, it is necessary to modify the regions close to the surface (“shell”) by doping with other chemical elements. This method will allow optimizing the physicochemical properties of the whole nanoparticle. In addition, a coating of the nanoparticles with an additional layer (“shell”) increases SPIONs’ resistance to oxidation and reduces their ability to agglomerate. In Ref. [72], γ-Fe_2_O_3_ nanoparticles coated with DBS (dodecyle benzene sulphonate) and CTAB (cetyltrimethyl ammonium bromide) were prepared by the microemulsion method. The microstructure of the nanoparticles was investigated by using synchrotron radiation X-ray spectroscopic methods (XPS, XANES, and EXAFS). The results of X-ray photoelectron spectroscopy showed the presence of chemical bonds between γ-Fe_2_O_3_ nanoparticles and surface-active groups. To study the interphase structure of SPIONs, the EXAFS method was used. EXAFS results showed the effect of the surface modification on the local atomic structure of the investigated nanoparticles. It was found that in DBS or CTAB coated samples an increase in the Fe-O bond lengths was observed as a result of structural distortions at the interface between γ-Fe_2_O_3_ and the coating created by the DBS or CTAB molecules. In Ref. [73], SPIONs coated with polymethyl silsesquioxane (PMSSO), which is a heat-resistant and oxidation resistant organo-silicon polymer, were studied. It was found that PMSSO in combination with superparamagnetic properties of iron nanoparticles can be used to develop biomedical materials. To obtain precise information on the positions of the silicon atoms and how the structure and the oxidation state of the iron atoms in these hybrid materials were changed by coating, both XANES and EXAFS methods were used. The experimental Fe K-edge XANES was compared with the spectrum of the reference γ-Fe_2_O_3_ compound. The result of XANES analysis showed that the chemical state of iron in the material is similar to the chemical state of iron in the maghemite crystal, i.e., the iron has an oxidation state of Fe^3+^ and the iron cations are surrounded by oxygen atoms in both octahedral and tetrahedral positions. Analysis of the Fourier transform of EXAFS spectra of the Fe K-edge gave information on the interatomic bonds length: direct Fe-Fe bonds, Fe-Fe contacts through oxygen (present in the ordered phases of iron oxide), and Fe ··· Si contacts (through oxygen). Based on the analysis of the EXAFS spectra, it was found that the iron atoms in these oxide nanoparticles are located in the same way as in maghemite crystals.

### 2.2. Mössbauer Spectroscopy

The phenomenon of nuclear gamma resonance [74,75] was discovered by R. Mӧssbauer in 1957. The recoil-free process of emission and absorption of γ-quanta made it possible to create a method for studying the structural and chemical state of materials—Mӧssbauer spectroscopy. Mӧssbauer spectroscopy has recently been increasingly used to study the local atomic structure and magnetic properties of iron oxide nanoparticles. Among a relatively small number of “Mössbauer-active elements”, iron occupies a special place. The largest number of Mössbauer studies are carried out on the isotope ^57^Fe, the natural content of which is ≈2% [76].

The ^57^Co in various metallic matrices is used as the source of γ-quanta in the ^57^Fe Mössbauer spectroscopy. The half-life of ^57^Co is 270 days. ^57^Co, as a result of electron capture, decays to ^57^Fe in the excited state with spin *I* = ±5/2. From the state with *I* = ±5/2 ^57^Fe passes with the emission of a γ-quantum of energy ≈136 keV into the excited state with *I* = ±3/2. And then to the ground state with *I* = ±1/2 emitting a γ-quantum of energy ≈ 14.4 keV. The last transition is used in ^57^Fe Mössbauer spectroscopy. As a rule, the energies in Mössbauer spectroscopy are given in mm/s, with 1 mm/s corresponding to 4.8 × 10^−8^ eV.

**Figure 4:**
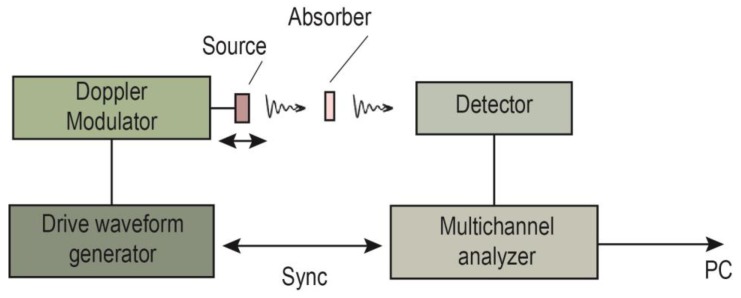
Scheme of the Mössbauer experiment in the transmission geometry.

Figure 4 shows the scheme of the Mössbauer experiment in the transmission geometry. The main part of any Mössbauer spectrometer is the Doppler modulator, which drives a γ-ray source or absorber. Movement is, as a rule, according to a triangular or sawtooth law. With the help of motion, due to the Doppler Effect, the γ-quantum energy is modulated in the required range. The γ-rays of the obtained energy range pass through the absorber (the sample under study), where they are partially absorbed resonantly. The unabsorbed γ-rays enter the detector.

The energy line of radiation from a γ-ray source is described by the Lorentz function. The energy absorption line has the same form. Thus, the Lorentz function is a form of the lines of Mössbauer spectra. A deviation from the Lorentz line shape gives information about the imperfection of the sample or indicates the shortcomings of the Mössbauer spectra fitting model. The main parameters of the Mössbauer spectra are isomer (chemical) shift (*δ*), quadrupole splitting (Δ), and hyperfine magnetic field on ^57^Fe (*H*) nuclei. The isomer shift is determined by the monopole contribution to the energy of the Coulomb interaction between the nucleus and the electrons. The value of δ is function of density of s-electrons:

(1) $$\delta =\frac{4\pi }{5}Ze^{2}\;\left[\;{\left|\Psi_{a}\left(0\right)\right|}^{2}-\;{\left|\Psi_{s}\left(0\right)\right|}^{2}\right]R\delta R$$

where *Z* is the atomic number, *e* is the electron charge, ${\left|\Psi_{a}\left(0\right)\right|}^{2}$ and ${\left|\Psi_{s}\left(0\right)\right|}^{2}$ are the electron densities inside the absorber and source nuclei, respectively, *R* is the nuclear radius, and *δR* = *R_e_* − *R_g_* is the difference between the mean radii of the excited and ground states of the nucleus. The isomeric shift is sensitive to the oxidation state and the coordination number of iron ions and, therefore, can be used to determine the oxidation states and the symmetry of the nearest environment [77].

The interaction between the electric quadrupole moment of the nuclear charge and the gradient of the electric field gradient in a non-spherically symmetric electric field splits the degeneracy of the excited state into two levels separated by the quadrupole splitting Δ:

(2) Δ=12 eQVZZ1+ η33

where *e* is the electron charge, *Q* is the quadrupole moment of the nucleus, *V_zz_* is the gradient of the electric field along the principal axis, *η* is the asymmetry parameter of the gradient electric field tensor. The Mӧssbauer spectrum in the presence of quadrupole splitting will consist of a doublet. The main cause of the quadrupole splitting is the gradient of the electric field created by the valence electrons of the atom itself and the neighboring atoms. The magnitude of quadrupole splitting can be an indicator of the asymmetric local environment of Fe^3+^ ions in the compounds under study [78]. Under a magnetic field (whether applied or internal), the nuclear levels of the excited state split into 2*I* + 1 levels. Transitions from the excited state to the ground state allowed by the selection rules (Δ*I* = 0, ±1) lead to the transformation of the ^57^Fe Mössbauer spectrum into a sextet. The distance between the first and sixth sextet lines makes it possible to determine the value of the hyperfine magnetic field on the ^57^Fe (*H*) nuclei. The value of *H* is determined by the chemical and structural properties of the material and allows one to characterize its magnetic properties.

Contrary to macroscopic magnetic particles, for which magnetic properties are mostly determined by long range ordering phenomena, in small and ultra small superparamagnetic nanoparticles like SPIONs, local magnetic characteristics start playing the most important role. Among various measuring techniques, Mössbauer spectroscopy represents an essential local tool for characterizing iron oxide nanoparticles. The advantage of the method is based on the extremely small natural width of the Mössbauer spectral lines. Local probing of Mössbauer spectroscopy allows one to get structural and magnetic information, such as atomic surroundings of iron atoms, precise oxidation state of iron ions and magnetic characteristics including the hyperfine magnetic field on the ^57^Fe nucleus. High elemental selectivity of Mössbauer spectroscopy makes it possible to identify the phases and their concentration in nanoparticles having multiple phases even if some phases are present in a very small amount. In addition, Mössbauer spectroscopy is sensitive to relaxation phenomena due to its short measuring time (≈10^−8^), thus it can be used to determine important magnetic characteristic such as the magnetic anisotropy energy constant (*K*). Study of SPIONs’ fine magnetic characteristics by Mössbauer spectroscopy is important to get deeper insight into the nature of the observed magnetic properties of these nanoparticles in the processes of local hyperthermia treatment and/or MRI diagnostics.

Mössbauer spectroscopy is widely used in studies of iron oxide nanoparticles. However, the small size of crystallites of nanoparticles is reflected in the structure of the Mössbauer spectrum [79]. In particular, a decrease in the size of crystallites leads to the appearance of superparamagnetism [80]. For superparamagnetic particles with a certain volume, *V*, there is a probability that the magnetization vector will spontaneously change its direction. The probability of such a process is proportional to exp(*KV*/*k_B_T*), where *K* is the magnetic anisotropy constant, *k_B_* is the Boltzmann constant, and *T* is the temperature. The relaxation time *t_sr_*, is time between two opposite directions of the magnetization vector. The *t_sr_* is determined by the expression [81]:

*t_sr_* = *t*_0_ exp(*KV*/*k_B_T*) (3)

where *t*_0_ is about 10^−11^с. To change the magnetization direction of the particle, it is necessary to overcome the energy barrier proportional to 2*KV*.

The presence of superparamagnetic relaxation leads to a “collapse” of the magnetic hyperfine structure of the Mössbauer spectrum, even at temperatures below the Neel temperature. This is due to the fact that the relaxation time *t_sr_* becomes less than the measurement time of the Mössbauer spectrum *t_m_* ≈ 10^−8^ s. The *t_sr_* decreases with lowering of temperature. At a certain temperature, called *T_B_* blocking temperature, at which *t_sr_* ≈ *t_m_*, a magnetic hyperfine structure appears on the Mössbauer spectrum. The *T_B_* value is defined as [82]:

*T_B_* = *aKV/k_b_* (4)

where *a* = 1/*ln*(*t_m_/t*_0_).

In addition, the average value of the magnetic hyperfine field decreases much more rapidly with increasing temperature, in comparison with single-crystal samples. This decrease is caused by the appearance of oscillations of the magnetization with respect to the direction corresponding to the minimum energy at *T* < *Т_B_*. The frequencies of such fluctuations usually far exceed the values of the Larmor frequency of the Mössbauer nuclei (≈10^8^ s^−1^). These collective magnetic excitations lead to a decrease in the magnetic hyperfine field, which in most cases can be approximated by the following Equation (5) [82]:

*H_exp_*(*T*) = *H*_0_(T) × (1 − *k_B_T*/2*KV*) (5)

where *H*_0_ is the hyperfine field in the absence of fluctuations. For *H*_0_, as a rule, the value of the hyperfine field of a massive sample is taken.

Expressions 4 and 5 allow one to estimate the magnitude of the magnetic anisotropy constant. If the value of *K* is known, for example, from measurements of the magnetic susceptibility, then the average size of the crystallites can be estimated.

**Figure 5:**
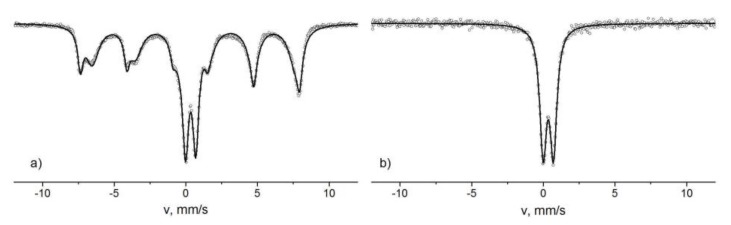
Mössbauer spectra of Fe_3_O_4_ nanoparticles d ≈ 13 nm (**a**) and γ-Fe_2_O_3_ d ≈ 8 nm (**b**) measured at room temperature.

**Figure 6:**
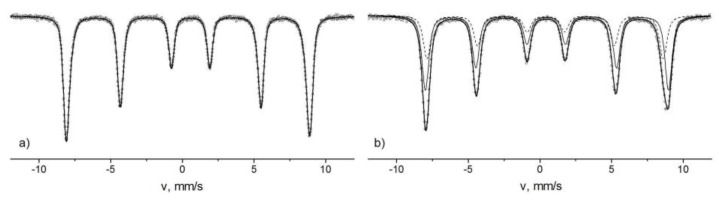
Mössbauer spectra of α-Fe_2_O_3_ nanoparticles d ≈ 30 nm (**a**) and γ-Fe_2_O_3_ (**b**) d ≈ 27 nm measured at 15 K.

**Figure 7:**
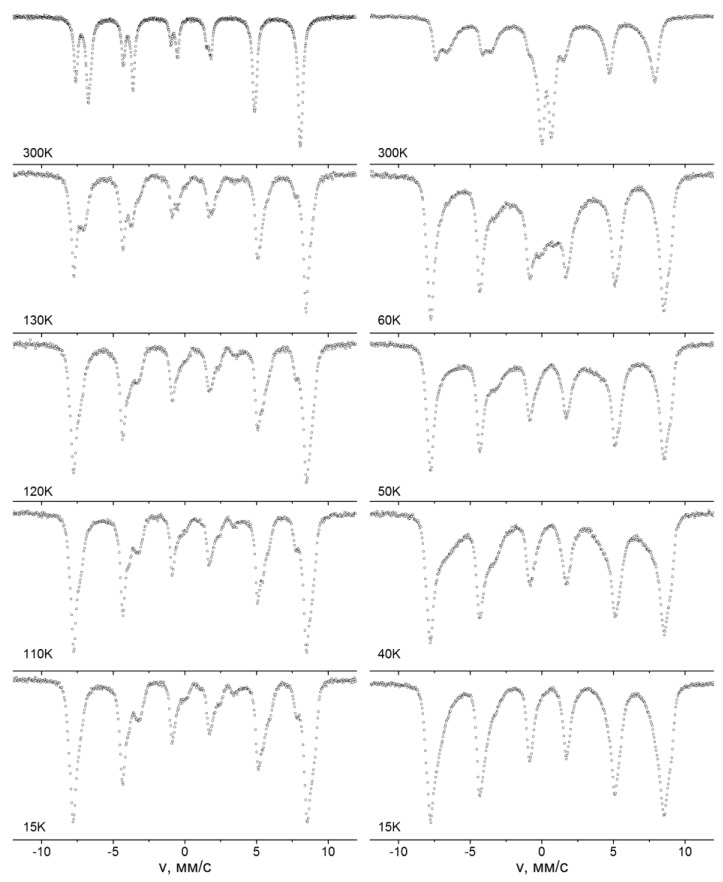
Mössbauer spectra of samples of Fe_3_O_4_ nanoparticles in the vicinity of the Verwey transition. The left column spectra of the particle sample d ≈ 32 nm, the right column spectra of the particle sample d ≈ 13 nm.

The influence of the superparamagnetic relaxation on the structure of the Mössbauer spectra complicates the model interpretation of the spectra and sometimes makes it impossible to identify the phases of the iron oxide nanoparticles. At temperatures above the blocking temperature, quadrupole splitting lines are observed on the spectra. The parameters of the Mössbauer spectra in this case are often practically identical. For example, the parameters of the doublets of the Mössbauer spectra of the hematite nanoparticles [83] and the goethite [84] with sizes less than 20 nm are approximately equal to each other. Another example is the spectra of Fe_3_O_4_ nanoparticles with diameter *d* ≈ 13 nm and γ-Fe_2_O_3_ with d ≈ 8 nm, shown in Figure 5. The spectrum of Fe_3_O_4_ particles consists of two sextets and a doublet, and the spectrum of γ-Fe_2_O_3_ has only the doublet. The values of the isomer shifts coincide and are equal to ≈0.34 mm/s. The quadrupole splitting is ≈0.70 mm/s. Thus, to fully identify the phases, it is often necessary to conduct low-temperature measurements. The lowering of the temperature leads to a decrease the effect of the superparamagnetic relaxation on the structure of the Mössbauer spectra [82]. In Figure 6, we show the spectra of α-Fe_2_O_3_ nanoparticles with diameter d ≈ 30 nm and γ-Fe_2_O_3_ with d ≈ 27 nm measured at the temperature of 15 K. The α-Fe_2_O_3_ spectrum is a sextet with *δ* ≈ 0.53 mm/s, which corresponds to Fe^3+^ ions in the octahedral oxygen environment. The γ-Fe_2_O_3_ nanoparticle spectrum contains asymmetric lines of magnetic splitting and consists of two sextets with *δ*_1_ ≈ 43mm/s and *δ*_2_ ≈ 0.52 mm/s. The values of the isomeric shift correspond to the Fe^3+^ ions in the tetrahedral and octahedral oxygen surroundings. Since γ-Fe_2_O_3_ has an inverse spinel structure, where iron ions occupy both tetrahedral and octahedral positions, the values of *δ* allow one to unambiguously determine the phase of the nanoparticles under study. The values of the sextet areas correspond to the concentrations of Fe ions in the occupied positions. The ratio of the sextet areas allows one to analyze the phase composition of mixtures of the different types of iron nanoparticles and determine the impurity phases. In the case of Fe_3_O_4_ particles, it is also necessary to take into account the process of Verwey exchange [85]. Fe_3_O_4_ has a reverse spinel structure, where the A-site is occupied by Fe^3 +^ ions, and the B-site is occupied by the Fe^3+^ and Fe^2+^ ions. Electron-exchange occurs between Fe^3+^ and Fe^2+^ ions of the octahedral sublattice. At room temperature, the time of this exchange is less than *t_m_*, therefore, on the spectrum at room temperature, one sextet is observed, with an isomeric shift value of about 0.68 mm/s corresponding to Fe ions in the intermediate charge state of “+2.5” (Figure 7), instead of two Fe^3+^ and Fe^2+^ sextets. When the temperature decreases, the electron transfer time increases, and at a certain temperature, Fe^3+^ and Fe^2+^ ions appear on the spectrum as separate components (Figure 7). The transition at which the Fe_3_O_4_ nanoparticles spectrum transformation takes place is called the Verwey temperature (T_V_). The presence of a component on the SPIONs Mӧssbauer spectrum corresponding to intermediate oxidation state (due to the Verwey exchange) is a reliable identifier of the Fe_3_O_4_ phase. In addition, a decrease in nanoparticle sizes leads to a decrease of T_V_ values. In Figure 6, we show the Mössbauer spectra of Fe_3_O_4_ nanoparticles with an average diameter of d ≈ 32 nm and d ≈ 13 nm at temperatures near the Verwey transition. The analysis of T_V_ value can also serve as a method for estimating the average particle size.

Thus, ^57^Fe Mössbauer spectroscopy, as a non-destructive method of physical and chemical identification, allows one to analyze the phase composition, the oxidation state, and local atomic structure around the Fe ions in the SPIONs and can also be used to determine the granulometric composition.

## 3. Conclusions

SPIONs’ local atomic structure, shape, and size are important parameters for tuning of the theranostic characteristics of these nanoparticles. XANES and Mössbauer spectroscopy are shown to be powerful tools for the analysis of the electronic and local atomic structure of these nanoparticles, especially in the case of small and ultra small sizes of the nanoparticles. XANES and Mössbauer spectroscopy as nondestructive tools could be an effective addition to standard laboratory methods for atomic structure determination like TEM and XRD. The possibility of high time resolution studies at the synchrotron radiation sources and X-ray free electron lasers in the near future will enable studies of not only the nanoparticles themselves at static conditions, but also their transformations during the processes in which they are involved.
